# Helmet noninvasive support for acute hypoxemic respiratory failure: rationale, mechanism of action and bedside application

**DOI:** 10.1186/s13613-022-01069-7

**Published:** 2022-10-14

**Authors:** Melania Cesarano, Domenico Luca Grieco, Teresa Michi, Laveena Munshi, Luca S. Menga, Luca Delle Cese, Ersilia Ruggiero, Tommaso Rosà, Daniele Natalini, Michael C. Sklar, Salvatore L. Cutuli, Filippo Bongiovanni, Gennaro De Pascale, Bruno L. Ferreyro, Ewan C. Goligher, Massimo Antonelli

**Affiliations:** 1 Department of Emergency, Intensive Care Medicine and Anesthesia, Fondazione Policlinico Universitario A. Gemelli IRCCS, Rome, Italy; 2 Istituto Di Anestesiologia E Rianimazione, Università Cattolica del Sacro Cuore Rome, Fondazione ‘Policlinico Universitario A. Gemelli’ IRCCS, L.go F. Vito, 00168 Rome, Italy; 3 Interdepartmental Division of Critical Care Medicine, University of Toronto, Toronto, Canada; 4 Department of Medicine, Division of Respirology, University Health Network/Sinai Health System, Toronto, Canada

## Abstract

**Introduction:**

Helmet noninvasive support may provide advantages over other noninvasive oxygenation strategies in the management of acute hypoxemic respiratory failure. In this narrative review based on a systematic search of the literature, we summarize the rationale, mechanism of action and technicalities for helmet support in hypoxemic patients.

**Main results:**

In hypoxemic patients, helmet can facilitate noninvasive application of continuous positive-airway pressure or pressure-support ventilation via a hood interface that seals at the neck and is secured by straps under the arms. Helmet use requires specific settings. Continuous positive-airway pressure is delivered through a high-flow generator or a Venturi system connected to the inspiratory port of the interface, and a positive end-expiratory pressure valve place at the expiratory port of the helmet;  alternatively, pressure-support ventilation is delivered by connecting the helmet to a mechanical ventilator through a bi-tube circuit. The helmet interface allows continuous treatments with high positive end-expiratory pressure with good patient comfort. Preliminary data suggest that helmet noninvasive ventilation (NIV) may provide physiological benefits compared to other noninvasive oxygenation strategies (conventional oxygen, facemask NIV, high-flow nasal oxygen) in non-hypercapnic patients with moderate-to-severe hypoxemia (PaO_2_/FiO_2_ ≤ 200 mmHg), possibly because higher positive end-expiratory pressure (10–15 cmH_2_O) can be applied for prolonged periods with good tolerability. This improves oxygenation, limits ventilator inhomogeneities, and may attenuate the potential harm of lung and diaphragm injury caused by vigorous inspiratory effort. The potential superiority of helmet support for reducing the risk of intubation has been hypothesized in small, pilot randomized trials and in a network metanalysis.

**Conclusions:**

Helmet noninvasive support represents a promising tool for the initial management of patients with severe hypoxemic respiratory failure. Currently, the lack of confidence with this and technique and the absence of conclusive data regarding its efficacy render helmet use limited to specific settings, with expert and trained personnel. As per other noninvasive oxygenation strategies, careful clinical and physiological monitoring during the treatment is essential to early identify treatment failure and avoid delays in intubation.

## Introduction

The role of non-invasive respiratory support in the management of acute hypoxemic respiratory failure (AHRF) is unclear, but evolving. Avoidance of intubation through noninvasive support improves patient outcomes by preventing the complications of invasive mechanical ventilation [1–3]. However, intubation is needed in a significant proportion of patients with AHRF treated with noninvasive support (30–60%), and is associated with higher mortality [4, 5]. This increased mortality may be due to delays in endotracheal intubation and the possible occurrence of patient self-inflicted lung-injury during the treatment [6–8].

The optimal balance between benefits and harms of preserving spontaneous breathing in patients with AHRF and/or acute respiratory distress syndrome (ARDS) is not fully understood [9]. For these reasons, recent guidelines have been unable to provide conclusive recommendations for facemask NIV in this setting [10]. In hypoxemic patients, noninvasive support can improve gas exchange and permit to avoid intubation in succeeding patients, but carries the risk of delaying intubation in patients failing the treatment. Delayed intubation worsens clinical outcome due to the occurrence of self-inflicted lung injury. Self-inflicted lung injury a form of injury similar to ventilator-induced lung injury, but mostly involving the dorsal, dependent lung zones and caused by the dysregulated inspiratory effort that severely hypoxemic patients may exhibit if spontaneous breathing is maintained [11–13].

NIV can be delivered through different interfaces, namely, oro-nasal masks, full-face masks, and helmets [14]. Most studies addressing the role of NIV during AHRF focused on oro-nasal and face masks [15]. Recently, there has been renewed interest towards the use of the helmet interface, mostly due to compelling results of systematic reviews and pilot clinical trials [2, 16–18]. Furthermore, a more thorough understanding of the physiology of spontaneous breathing during AHRF and ARDS highlighted the possible role of specific ventilator settings that can be delivered through the helmet interface and can potentially mitigate the risk of self-inflicted lung injury. These essentially include the possibility to provide higher levels of positive end-expiratory pressure (PEEP) for prolonged periods without interruptions [19, 20].

In this narrative review, we discuss the physiological rationale for the use of helmet support as first-line treatment of AHRF/ARDS, and we describe the technicalities for its safe application in hypoxemic patients.

## Methods

This narrative review was based on a systematic search of the medical literature, which was performed according to a protocol published in PROSPERO (CRD42020201563). We performed a computerized search of MEDLINE, PubMed, Embase and the Cochrane Central Register of Controlled Trials (CENTRAL) database for relevant English-language studies from inception to June 2021. Most relevant studies published up to August 2022 were subsequently included. Study inclusion for our review included any observational study, interventional trial or reviews on adults with AHRF treated with helmet NIV or describing the physiological effects of spontaneous breathing during hypoxemic respiratory failure. We included studies describing (1) how to set up helmet support, (2) its physiological effects, (3) ventilator settings capable of limiting lung injury during spontaneous breathing and (4) clinical outcomes of patients receiving helmet support, with or without a comparison to other noninvasive oxygenation strategies. Two independent reviewers performed an initial screening of all retrieved papers by title and abstract. Then, full-text screening was performed. At any stage, when discussion was unable to reach a definitive conclusion, disagreements were solved by a third reviewer.

Among 510 citations, a total of 100 studies, including 8 randomized trials and three meta-analyses, were included.

## Spontaneous breathing in hypoxemic respiratory failure

### Non-invasive respiratory support—a double-edged sword

In patients with AHRF in intensive care unit (ICU), maintenance of spontaneous breathing avoids sedation and passive ventilation, thereby limiting diaphragm dysfunction and delirium, facilitating mobilization, and reducing the risk of ventilator-associated complications (e.g., ventilator-associated pneumonia, ICU-acquired weakness) [21–23]. Moreover, spontaneous breathing improves aeration of dependent lung regions and redistributes pulmonary blood flow [24, 25], finally improving ventilation/perfusion matching and oxygenation [26, 27].

Preserving spontaneous breathing with noninvasive support may yield, however, risks related to delays in endotracheal intubation, with detrimental effects on mortality [4, 28, 29]. Patients who fail NIV exhibit elevated inspiratory effort, leading to self-inflicted lung injury and load-induced injury to the diaphragm [6, 30, 31]. High inspiratory effort generates tidal volumes beyond the safe thresholds of lung protection, which can be further exacerbated by the inspiratory assistance of pressure support [32–34].

### Mechanisms of injury from spontaneous breathing and the role of PEEP

In critically ill patients with AHRF, respiratory drive and inspiratory effort are increased by lung injury, high alveolar dead space, reduced pulmonary compliance, increased neural ventilatory response to carbon dioxide (CO_2_), and higher CO_2_ production by respiratory muscles [13]. This leads to increased activation of respiratory muscles, which may not be capable of matching the brain’s desired CO_2_ clearance [11, 12]. Several mechanisms explain why elevated respiratory effort may be injurious in patients with AHRF. High inspiratory effort translates into large swings in transpulmonary pressure and high tidal volumes, that yield high lung stress and strain [27, 35]. Overinflating the normally aerated lung tissue, which is markedly reduced because of inflammatory edema (i.e., the baby lung), leads to lung injury and multi-organ failure [36–38].

Atelectasis and consolidation are not distributed homogeneously in the lung [39, 40]. Thus, the inflationary forces generated by diaphragmatic contraction are not uniformly transmitted throughout the tissue. In terms of mechanical response to distending stress, collapsed, dependent dorsal lung regions are likely to demonstrate ‘solid-like’ rather than ‘fluid-like’ behaviour. As a result, an alveolar pressure gradient develops between the different lung zones leading to a ‘pendelluft’ phenomenon, which is an intra-tidal displacement of gas from non-dependent (normally aerated regions with a liquid-like behaviour) to dependent lung regions (solid-like behavior) in the early phase of inspiration [41]. Dorsal lung regions are, therefore, more distended than ventral lung regions and subject to additional overstretch, perpetuating lung injury. This pendelluft phenomenon is largely dependent on the intensity of inspiratory effort, and can result in hidden, local overstretch of the dependent lung even if global transpulmonary pressure swings and inspired tidal volume are within a safe range [42, 43].

Increased lung perfusion and hydrostatic edema can be magnified by the high transvascular pressure produced by intense negative swings in pleural pressure: this generates negative-pressure pulmonary edema, further aggravating lung injury [44–46].

The diaphragm is also injured by intense inspiratory effort, leading to diaphragm myotrauma and diaphragm dysfunction, which detrimentally affects clinical outcome [47].

Strategies to directly reduce inspiratory effort (e.g., correction of metabolic acidosis, treatment of fever, analgesia and sedation) and the application of high PEEP levels may mitigate the risk of lung injury due to dysregulated inspiratory effort.

**Fig. 1:**
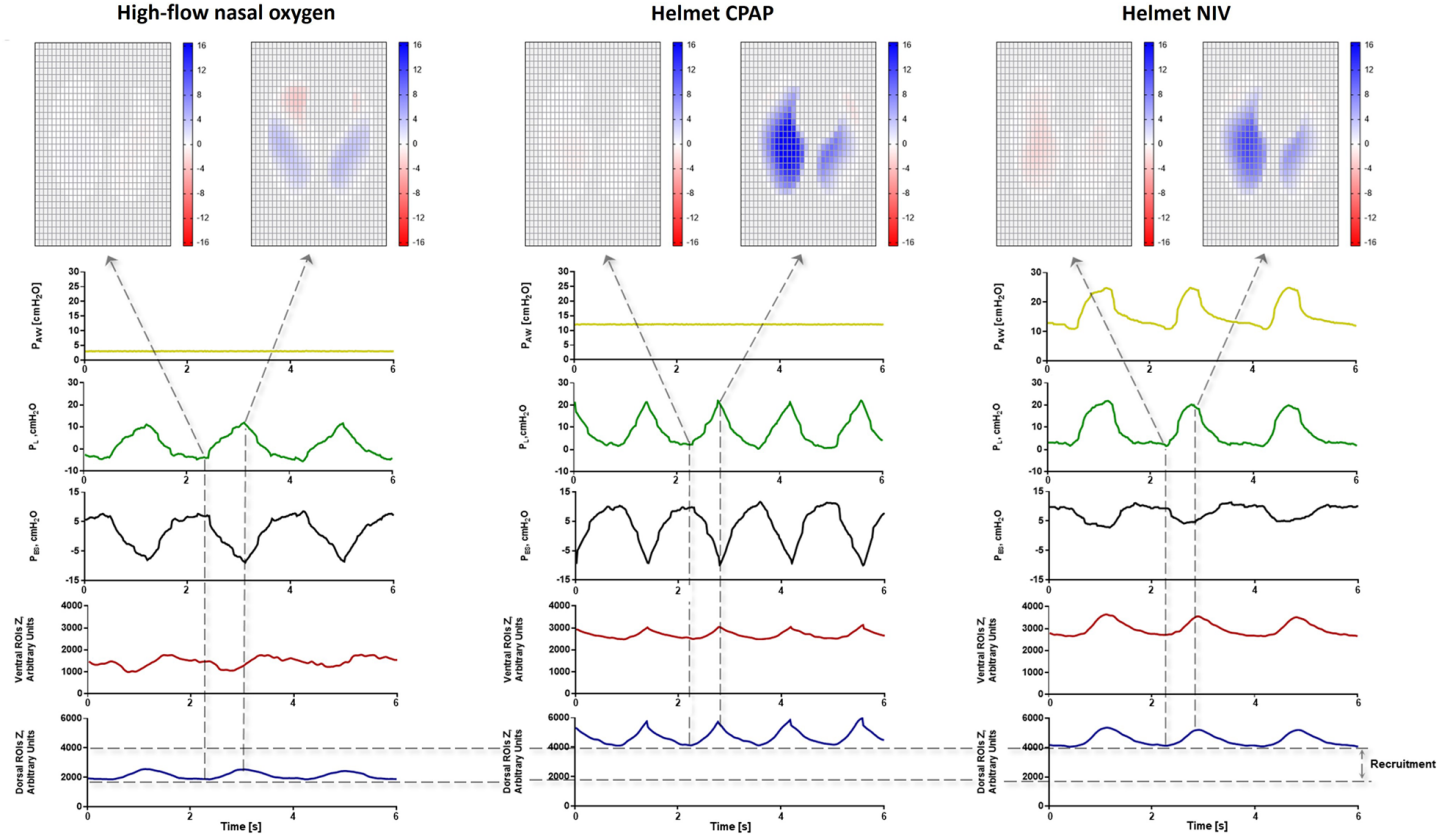
Comparison of representative tracings of airway pressure, transpulmonary pressure esophageal pressure and global and regional electrical impedance tomography during spontaneous breathing with high-flow nasal, helmet CPAP and NIV in a patient with severe hypoxemic respiratory failure. The left panel shows the respiratory mechanics during spontaneous breathing with high flow oxygen mask. Due to the high inspiratory effort and to the inhomogeneity of the lung, it is possible to appreciate the Pendelluft effect. The start of inspiration (marked by the initial negative deflection of the Pes) is coincident with the increase of electrical impedance tomography in the Global ROI tracing (∆Z, %). However, while in the dorsal regions of the lungs (dependent regions) there is an increase of ∆Z%, in the ventral region there is a decrease of ∆Z% (non-dependent regions). This represents the “Pendelluft effect”, an intra-tidal displacement of air from non-dependent to dependent lung regions, causing local overstretch of the latter. The first dotted line marks the moment when the ∆Z% signal in the most ventral ROI stops decreasing and local inflation begins. In right panels, the respiratory mechanics of the same patient receiving helmet CPAP and pressure support are shown. High PEEP generates recruitment in dorsal lung regions and mitigate the pendelluft effect and enhances more homogeneous lung inflation. Presence of pressure support causes a decrease of the inspiratory effort ∆Pes swing. Heat maps describe lung regional inflation (blue pixels) and deflation (red pixels). In the absence of PEEP, a significant pendelluft effect is documented (red pixels during inspiration), which reflects the intra-tidal shift of gas from anterior non-dependent lung regions to posterior dependent lung regions. This is abolished by high PEEP delivered through the helmet interface, which makes inflation homogenous across the whole lung tissue. Acronyms: PAW, airway pressure; PES, esophageal pressure; ∆Z %, electrical impedance tomography signal variation; ROI, region of interest; VV, ventral-ventral; MV, middle-ventral; MD, middle-dorsal; DD, dorsal-dorsal

PEEP-induced alveolar recruitment improves hypoxemia and may improve the homogeneity of inflation across the different lung regions [48, 49]. High PEEP (10–15 cmH_2_O) favours a more homogeneous distribution of inspiratory pressure across the lung tissue, thus reducing pendelluft (Fig. 1) and progression of lung injury; it also leads to neuromechanical uncoupling and reduces inspiratory effort, tidal volume and transpulmonary driving pressure, even if the neural stimulus remains unchanged [20, 50, 51].

In summary, application of moderate-to-high PEEP may be essential to minimize the risk of self-inflicted lung injury in spontaneously breathing AHRF and ARDS, especially in case of moderate-to-severe hypoxemia (PaO_2_/FiO_2_ < 200 mmHg) [27]. During facemask NIV, PEEP ranging between 5 and 8 cmH_2_O are usually applied [52], while higher values are difficult to achieve because of air leaks and patient discomfort [18]. By contrast, the helmet interface allows delivery of moderate-to-high PEEP (10–15 cmH_2_O) for prolonged treatments with good tolerability and without significant leaks.

## Helmet support

The Helmet is a transparent hood that covers the entire head of the patient with soft neck collar that allows the system to seal at the patient’s neck. The interface is further secured by straps under the arms. At least 2 ports are present, which are connected to separate tubes for inhaled and exhaled gas, respectively. All commonly used helmets are latex-free and available in multiple sizes.

Helmet interface may be used to deliver either continuous positive airway pressure (CPAP, i.e., the sole application of PEEP without any inspiratory pressure support) or NIV in pressure support mode (PSV). For the same PEEP level, the major difference between CPAP and NIV in the capability of the latter to best reduce inspiratory effort. From a theoretical standpoint, in hypoxemic patients, CPAP could be preferred in case the inspiratory effort before treatment start is low (< 10 cmH_2_O), while NIV mostly benefits patients with high inspiratory effort (> 10 cmH_2_O) [53].

**Fig. 2:**
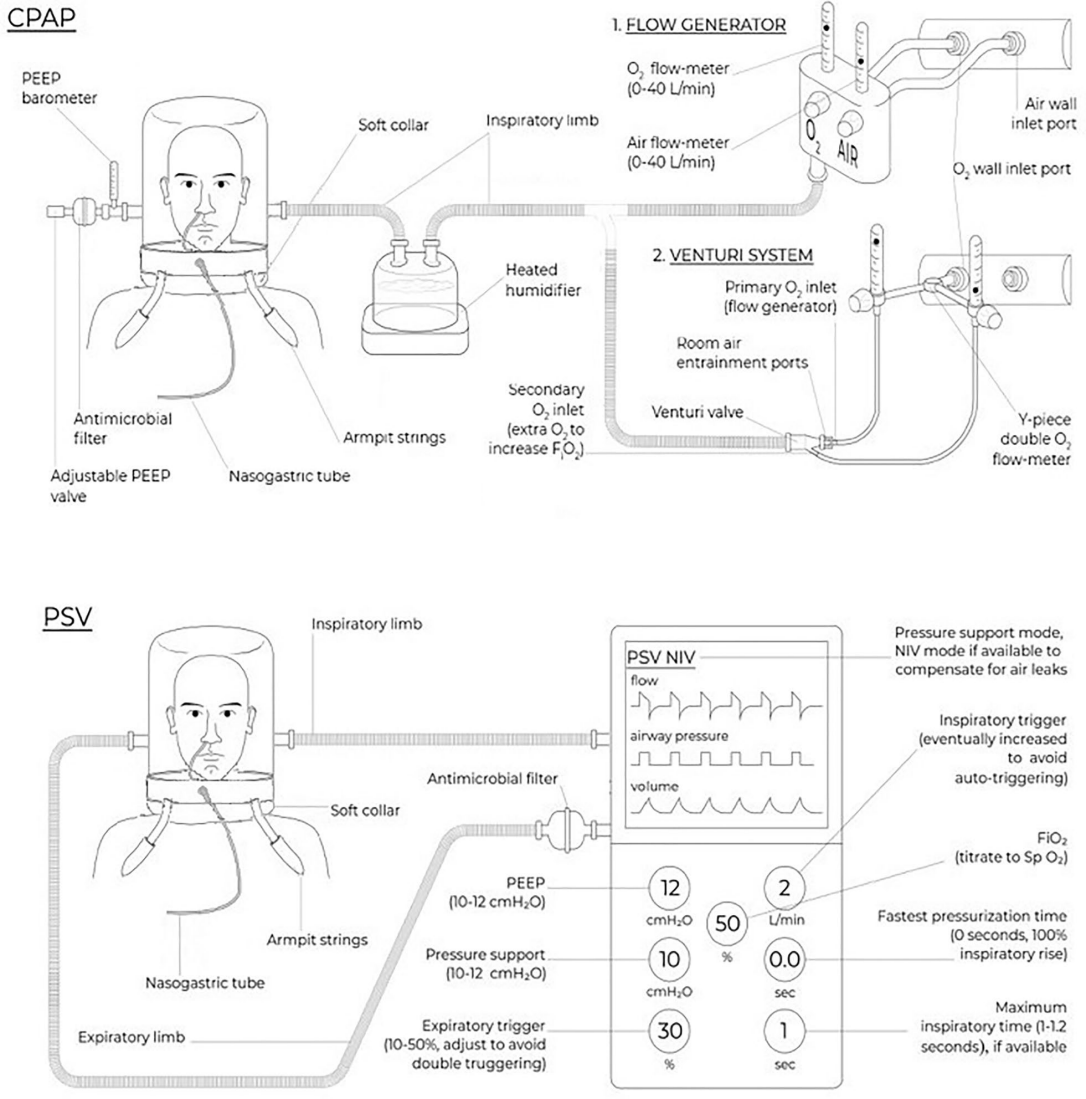
Helmet interface and circuit set-up for CPAP and NIV. The helmet has a transparent hood and a soft collar that contacts the body at the neck and/or shoulders. It covers the head and neck without making direct contact with the patient’s face and it is fixed around the axillae. At least 2 ports are present, which are usually connected to two separate tubes for inhaled and exhaled gas (double-tube circuit). An antibacterial filter should be placed on the expiratory port

Given the unique characteristics of the interface, specific settings are required to optimize the treatment: these are described in Table 1. Circuit set-up is displayed in Fig. 2.

**Table 1:** Helmet settings in patients with acute hypoxemic respiratory, with and without pressure support

Ventilatory setting	Helmet NIV	Helmet CPAP
Ventilatory circuit	Ventilator with bitube circuit and antimicrobial filter on expiratory port	High flow generator with PEEP valve and antimicrobial filter on expiratory port
PEEP	10–15 cmH_2_O	10–15 cmH_2_O
Pressure support	10–14 cmH_2_O	–
Fresh gas flow	–	50–60 L/min
FiO_2_	Titrated to obtain SpO_2_ ≥ 92% and ≤ 98%	Titrated to obtain SpO_2_ ≥ 92% and ≤ 98%
Pressurization rate	0.00 s (or fastest possible pressurization rate)	–
Inspiratory flow trigger	2 L/min or 2 cmH_2_O	–
Cycling	10–50% of maximum inspiratory flow	–
Maximum inspiratory time	1.2 s	–
Gas conditioning	No humidification needed if minute ventilation < 35 L/min	Active heating and humidification (37 °C or 34 °C according to patient’s comfort)

## Specific settings

### CPAP

Theoretically, helmet CPAP can be delivered through a mechanical ventilator or by an adjustable continuous flow-generator in combination with a PEEP valve [54]. Ventilator-delivered helmet CPAP may be inherently unsafe, since the absence of inspiratory pressure support leads to a total system minute ventilation (washout flow) significantly lower than the 30–50 L/min needed to avoid CO_2_ rebreathing [55–57]. For this reason, a high-flow generator or a VenturiSystem providing 50–60 L/min of flow and a PEEP valve (10–15 cmH_2_O) represent the safest set-up to deliver helmet CPAP. In this setting, the application of a heated humidifier is needed, since fresh gas flows > 40 L/min would otherwise lead to under-humidification inside the helmet [58–60].

### PSV-NIV

The main helmet-specific PSV settings are [13, 17, 61–64]:

#### Circuit set-up

Double-limb ventilators should be used to provide helmet NIV. Both modern high-performance turbine ventilators and gas-compressed ventilators can be used, with the latter being preferable. A double-tube circuit should be preferred over a Y-piece circuit, in terms of ventilator pressurization performance, patient-ventilator interaction and avoidance of CO_2_ rebreathing.

#### Higher PEEP level (10–15 cmH_2_O)

Increasing PEEP reduces interface compliance, thus minimizing the amount of pressure support wasted to pressurize the interface and reducing airway pressurization time. Importantly, increasing PEEP contributes to reduce air leaks by abutting the helmet against the patient's shoulders.

#### Higher pressure support (10–14 cmH_2_O)

Increasing pressure support further reduces helmet compliance reducing the amount of pressure wasted to pressurize the interface and ensuring adequate inspiratory support to unload the respiratory muscles. Moreover, a higher-pressure support generates a higher washout flow, which is crucial to avoid CO_2_ rebreathing.

#### Fastest pressurization rate

This aims at minimizing the under-assistance of respiratory muscles during the peak inspiratory effort. Vargas and colleagues demonstrated that increasing PEEP and pressure support by 50% and use of the fastest pressurization rate significantly improved the unloading of respiratory muscles.

#### Gas conditioning

Gas conditioning by either heated humidifiers or heat and moisture exchangers to reach a minimum absolute humidity of 15 mgH_2_O/L is recommended during facemask NIV [65–68]. However, these data cannot be generalized to the helmet interface. Preliminary data seem to show that no humidification is needed during helmet NIV if the total system’s minute ventilation does not exceed a threshold of around 40 L/min, which is the case for hypoxemic patients treated with helmet NIV. A double-tube circuit without any humidification reduces discomfort and provides sufficient conditioning of the inspired gas, without any effect on inspiratory effort and work of breathing [69].

### Specific features

#### Internal volume, dead space and CO_2_ rebreathing

The internal volume of the helmet is much larger than any other NIV interface (around 18 L) and it behaves as a semi-closed mixing chamber. As such, some of the patient’s exhaled gas is not eliminated from the helmet and instead mixes with fresh gas coming from the inspiratory limb of the circuit, possibly resulting in CO_2_ rebreathing [56, 70–72]. CO_2_ concentration inside the helmet depends on the balance between the patient’s CO_2_ elimination and the system’s washout flow. Consequently, high fresh gas flows are needed to avoid rebreathing (flow rates of at least 30–50 L/min have been shown to be necessary for this purpose) during CPAP [55], and pressure support of 12 cmH_2_O is usually efficient to avoid the risk of clinically relevant CO_2_ rebreathing during NIV [62]. During NIV, the use of a bi-tube circuit enables CO_2_ washout by ventilator expiratory flow-by, that can reach 15 L/min in modern gas-compressed mechanical ventilators provided with a NIV-dedicated module.

#### Physiological effects of helmet NIV

**Fig. 3:**
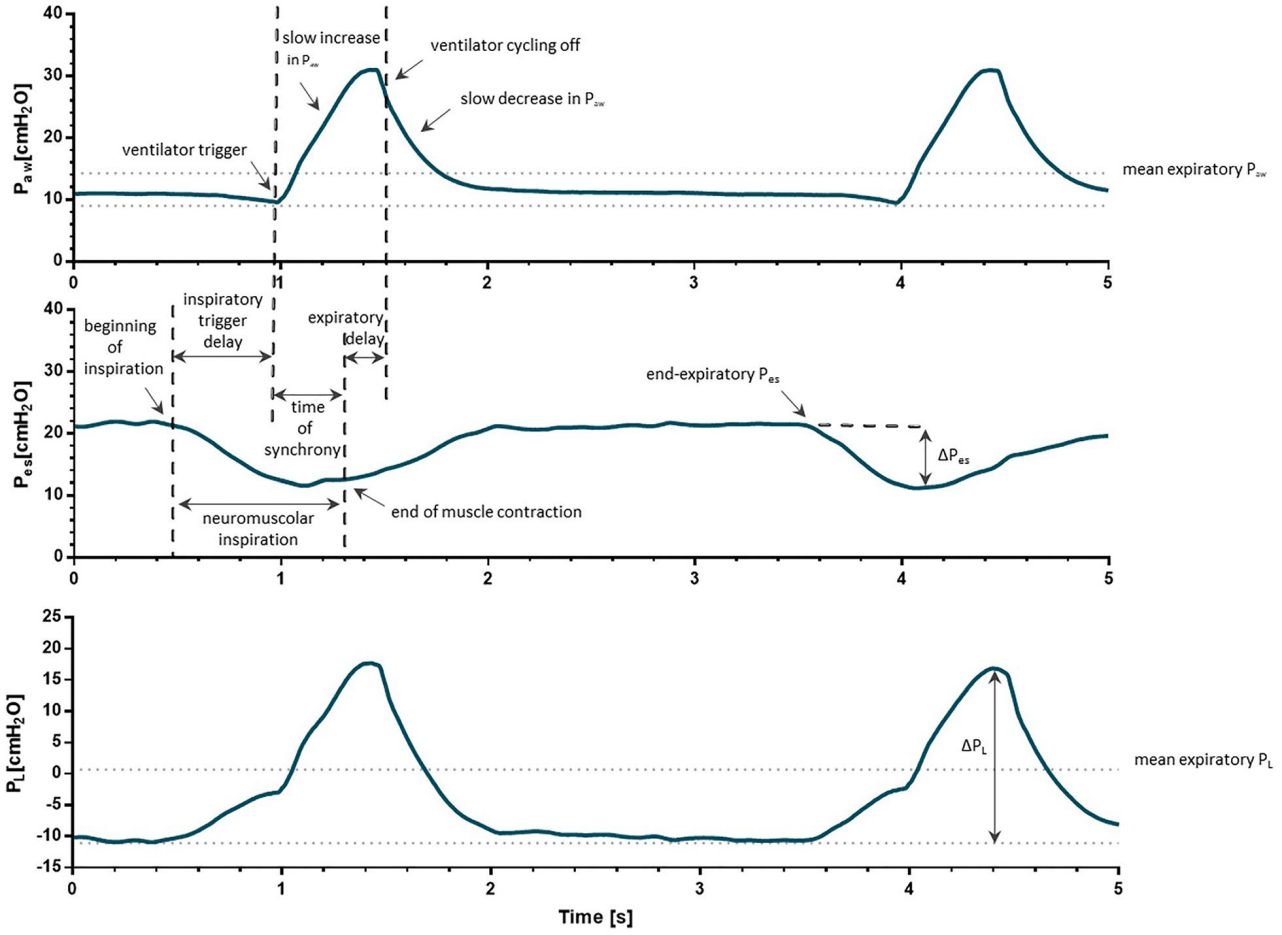
Representative tracings of respiratory mechanics of a patient treated with helmet pressure support ventilation. Due to the high compliance of the interface, asynchronies are common during helmet NIV. Inspiratory and expiratory trigger delays are displayed, together with the slow increase and decay in airway pressure. Despite the short time of synchrony, the mean expiratory airway pressure is higher than the set PEEP (dotted lines in the Paw tracing) and the mean expiratory transpulmonary pressure is higher than the end-expiratory transpulmonary pressure (dotted lines in the PL tracing). Due to the significant trigger delays caused by interface compliance, inspiratory effort and ventilator assistance are (at least in part) out-of-phase, avoiding excessive dumps in transpulmonary pressure during inspiration. This de-synchronization may further enhance lung protection. Acronyms; PES, esophageal pressure; PL, transpulmonary pressure

During NIV, inspiratory pressurization is slower than with mask interfaces due to significant trigger delays (0.1–0.5 s) and because part of the pressure is dissipated to distend the interface. Similarly, pressure decay after cycling off is slower and delayed, often leading to patient’s expiration against a positive pressure which is higher than the set PEEP (this represents an additional resistance to patient’s expiratory flow, it might contribute to enhanced alveolar recruitment) [62]. Inspiratory desynchronization and patient-ventilator asynchronies, although formal and common during helmet NIV, do not lead to discomfort, as the patient is able to inhale/exhale in the reservoir of the interface [62]. Inspiratory de-synchronization may exert lung-protective effects, as inspiratory effort and pressure support are in part out-of-phase, finally limiting the amplitude of transpulmonary pressure inspiratory swings [73, 74] (Fig. 3).

As compared to high-flow nasal oxygen, helmet NIV improves oxygenation and reduces inspiratory effort and dyspnoea without changes in PaCO_2_, comfort, or transpulmonary pressures. Patients with lower inspiratory effort during high-flow nasal oxygen can develop increased transpulmonary pressures on helmet NIV, while patients with higher effort during high-flow nasal oxygen show the most relevant reduction in transpulmonary pressure swings on helmet NIV.

Taken together, these data indicate that helmet NIV might have advantages over high-flow nasal oxygen in most severely hypoxemic patients, especially among those exhibiting intense inspiratory effort, perhaps because of the higher achievable PEEP levels with their attendant benefits in terms of alveolar recruitment and reducing inspiratory effort. Conversely, helmet NIV may increase transpulmonary pressures in patients with low inspiratory effort, since the increase in delivered pressure support is not offset by a clinically relevant decrease in negative swings of pleural pressure. In this latter subgroup, the use of a lower level of pressure support or CPAP may help mitigate the increase in transpulmonary pressure.

#### Monitoring

As per any other noninvasive oxygenation strategy, helmet support should be used under strict clinical and physiological monitoring. Careful monitoring is needed to promptly identify treatment failure, and not to delay endotracheal intubation and the institution of protective ventilation [75–77].

Clinically, worsening hypoxemia, increased respiratory rate, lack of dyspnea relief are all factors that should prompt the decision to intubate patients [17, 78–80].

Given the mechanical characteristics of the helmet interface, such as the inability to transiently occlude the airway, some of the non-invasive validated methods to assess inspiratory effort and drive (occlusion pressure, P_0.1_) may not be reliable [81, 82]. The monitoring of inspiratory effort in theory may help identify patients with a higher likelihood of self-inflicted lung injury and should prompt strategies to reduce this risk. While several indices of respiratory drive and effort exist, these are difficult to evaluate in nonintubated patients. Commonly measured parameters as respiratory rate and dyspnea are inaccurate measures of inspiratory effort, which is measured by esophageal manometry through the negative deflection of esophageal pressure during inspiration [33, 34, 83]. This minimally invasive method is an advanced monitoring technique achieved with nasogastric tube equipped with an esophageal balloon to measure esophageal pressure, which reflects pleural pressure. Esophageal manometry allows inspiratory effort and transpulmonary pressure measurement, assessment of the work of breathing, detection of patient-ventilator asynchronies and, possibly, titration of noninvasive support to personalize protective ventilatory settings. Inspiratory effort persistently greater than 10 cmH_2_O during NIV, both delivered with facemask and helmet, is strongly associated with the subsequent need for endotracheal intubation, suggesting that inspiratory effort monitoring may play a crucial role in assessing the risk of self-inflicted lung injury during helmet NIV [33, 34, 62].

Patients with high severity of illness (Simplified Acute Physiology Score II > 34), older age, or those who fail to improve PaO_2_/FiO_2_ or maintain persistently high inspiratory effort after 1 h of treatment are at higher risk of failure [75]. Validated clinical scores such as the ROX index (ratio of SpO_2_/[FiO_2_*respiratory rate]) and the HACOR scale (heart rate, acidosis, consciousness, oxygenation and respiratory rate) have been used to early predict failure during high-flow nasal oxygen and facemask NIV, respectively [84, 85]. Their reliability under helmet support, although physiologically sound, is undemonstrated.

With standard equipment, tidal and minute ventilation cannot be reliably monitored during helmet support, since a substantial portion of the tidal volume inflates the helmet and does not reach the patient. During PSV, minute ventilation displayed by the ventilator represents the system’s washout flow.

## Benefits related to helmet interface

Aside from the physiological benefits, the helmet interface offers several practical advantages over other interfaces. First, it allows the patient to see, read, interact with the environment, it facilitates coughing, improves overall comfort, and can facilitate early mobilization and physiotherapy. It also allows the patient to drink though a specific straw independently. High tolerability allows continuous treatment, reducing or eliminating the need for intermittent sessions, that are unavoidable during facemask NIV [78]. It can be applied to any patient regardless of the face contour and allows the application of higher PEEP levels without relevant air leaks or ocular irritation.

## Outcomes

A summary of the clinical studies comparing helmet support with other techniques is provided in Table 2. Notably, most of the studies were conducted in Italy.

**Table 2:** Comparative studies regarding helmet support

Publication	PMID	Study design	Setting	Patient Population	Helmet treatment	Control treatment	Intubation Rate	Mortality Rate	Main finding	Secondary findings
Antonelli et al. (2002)	11990923	Case control prospective study	ICU + emergency room	AHRF Helmet PSV group mean PaO_2_/FiO_2_ 125 mmHg Face mask PSV group mean PaO_2_/FiO_2_ 124 mmHg	Helmet PSV group (*n* = 33)	Face mask PSV group (*n* = 66)	Helmet PSV group 24% Face mask PSV group 32%	Helmet PSV group 9% Face mask PSV group 26%	Helmet NIV was as effective as face-mask NIV	Helmet improves tolerance, allows prolonged treatments and reduces complications related to skin ulcers
Principi et al. (2003)	14593457	Prospective clinical study	Hematological ward	AHRF in hematological malignancy patients Helmet CPAP group mean PaO_2_/FiO_2_ 135 mmHg Face mask CPAP group mean PaO_2_/FiO_2_ 140 mmHg	Helmet CPAP group (*n* = 17)	Face mask CPAP group (*n* = 17)	Helmet CPAP group 0% Face mask CPAP group 41%	Helmet CPAP group 23% Face mask CPAP group 47%	Helmet CPAP was better tolerated than face mask CPAP, avoiding skin breakdown and allowing a longer period of continuous treatment with fewer ETI	
Rocco et al. (2004)	15539720	Case control study	ICU	AHRF in immunocompromised patients Helmet PSV group mean PaO_2_/FiO_2_ 109 mmHg Face mask PSV group mean PaO_2_/FiO_2_ 101 mmHg	Helmet PSV group (*n* = 19)	Face mask PSV group (*n* = 19)	Helmet PSV group 37% Face mask PSV group 47%	Helmet PSV group 31% Face mask PSV group 47%	Helmet NIV was as efficient as face mask NIV in avoiding ETI and improving gas exchange	
Cosentini et al. (2010)	20154071	Multicenter randomized controlled trial	Emergency department	Mild AHRF in community-acquired pneumonia Helmet CPAP group mean PaO_2_/FiO_2_ 249 mmHg Standard oxygen therapy (Venturi mask) mean PaO_2_/FiO_2_ 246 mmHg	Helmet CPAP group (*n* = 20)	Standard oxygen therapy (Venturi mask) group (*n* = 27)	Helmet CPAP group 0% Standard oxygen group 0%	Helmet CPAP 0% Standard oxygen 0%	CPAP delivered by helmet more efficiently improves oxygenation at 1 h	
Squadrone et al. (2010)	20533022	Single-center randomized controlled trial	Hematological ward	Prevention of ARDS in patients with hematological malignancy Helmet CPAP group mean PaO_2_/FiO_2_ 441 mmHg Standard oxygen therapy (Venturi mask) mean PaO_2_/FiO_2_ 392 mmHg	Helmet CPAP group (*n* = 20)	Standard oxygen therapy (Venturi mask) group (*n* = 20)	Helmet CPAP group 10% Standard oxygen therapy (Venturi mask) group 40% [95% CI 0.29–0.85]	Helmet CPAP group 15% Standard oxygen therapy (Venturi mask) group 75%	Early CPAP in immunosuppressed patients with hematological malignancy may prevent evolution to ARDS requiring ventilatory support and ICU admission	
Brambilla et al. (2014)	24817030	Multicenter randomized controlled trial	High dependency Units	AHRF Helmet CPAP group mean PaO_2_/FiO_2_ 134 mmHg Standard oxygen therapy (Venturi mask) group mean PaO_2_/FiO_2_ 148 mmHg	Helmet CPAP group (*n* = 40)	Standard oxygen therapy (Venturi mask) group (*n* = 41)	Met prespecified ETI criteria: Helmet CPAP group 15% Standard oxygen therapy (Venturi mask) group 63% [95% CI 0.11–0.51] ETI: Helmet CPAP group 5% Standard oxygen therapy (Venturi mask) group 2%	Helmet CPAP group 5% Standard oxygen therapy (Venturi mask) group 17%	Helmet CPAP reduces the risk of exhibiting objective criteria leading to endotracheal intubation	Helmet CPAP group yielded faster improvement in PaO_2_/FiO_2_ ratio, respiratory rate and respiratory distress
Patel et al. (2016)	27179847	Single-center randomized clinical trial	ICU	ARDS Face mask NIV mean PaO_2_/FiO_2_ 144 mmHg Helmet NIV mean PaO_2_/FiO_2_ 118 mmHg	Helmet NIV group (*n* = 44) PEEP 8 cmH2O (5.0–10.0) Pressure support 8 cmH2O (5.6–10.0)	Face mask NIV group (*n* = 39) PEEP 5.1 cmH2O (5.0–8.0) Pressure support 11.2 cm H2O (10.0–14.5)	Face mask NIV group 62% Helmet NIV group 18% [95% CI − 62 to − 24]	Face mask NIV group = 56% Helmet NIV group = 34% [95% CI − 43 to − 1]	Helmet NIV was associated with a reduction of intubation rates compared to delivery by face mask	Helmet NIV reduces 90-day mortality and ICU length of stay
Liu et al. (2020)	33293689	Single-center randomized controlled trial	ICU	AHRF in chest trauma Helmet NIV group mean PaO_2_/FiO_2_ 163 mmHg Face mask NIV group mean PaO_2_/FiO_2_ 162 mmHg	Helmet NIV group (*n* = 29)	Face mask NIV group (*n* = 30)	Helmet NIV group 3% Face mask NIV group 10%	Helmet NIV group 3% Face mask NIV group 3%	Helmet NIV decreased complications related to NIV, increased PaO_2_/FiO_2_ and improved tolerance compared with face mask NIV	
Gaulton et al. (2020)	32984836	Retrospective multicenter study	ICU	COVID-19 AHRF in patients with mean BMI kg/m2 = 35.5 SpO_2_ < 92% with 6 L/min nasal cannula	Helmet CPAP group (*n* = 17)	HFNO group (*n* = 42)	ETI within 7 days of treatment: Helmet CPAP group 18% HFNO group 52%	Death at 7 days: Helmet CPAP group 6% HFNO group 19%	Adjusting for age, helmet CPAP was associated with a decreased odds of intubation	In obese patients Helmet CPAP is effective in reducing the ETI rate
Grieco et al. (2021)	33764378	Randomized multicenter clinical trial	ICU	COVID-19 AHRF Helmet NIV mean PaO_2_/FiO_2_ 105 mmHg HFNO mean PaO_2_/FiO_2_ 102 mmHg	Helmet NIV group (*n* = 54) Continuous treatment PEEP 12 (10—12) Pressure Support 10 (10–12)	HFNO group (*n* = 55)	Helmet NIV 30% [95% CI 19–43] HFNO 51% [95% CI 38–64]	Helmet NIV = 24% [95% CI 15–37] HFNO = 25% [16 to 38]	Helmet NIV + HFNO or HFNO alone do not affect respiratory support free days	Helmet NIV reduces rate of ETI and increases invasive VFD at day 28
Rezoagli et al. (2021)	34,091,270	Single-center observational retrospective study	ICU	AHRF Mean PaO_2_/FiO_2_ of all patients 157 mmHg	Helmet CPAP group (*n* = 51)	Face mask NIV group (*n* = 18)	Helmet CPAP 29% Face mask NIV 53% [95%CI]	ICU mortality: NIV success 1% NIV failure 22%	The use of Helmet CPAP compared to face mask NIV was an independent predictor of noninvasive respiratory support success	A positive fluid balance was independently associated with a significant increase of intubation
Colaianni-Alfonso et al. (2022)	36049548	Prospective cohort study	ICU	COVID-19 AHRF Helmet CPAP mean PaO_2_/FiO_2_ 96 mmHg Face mask CPAP mean PaO_2_/FiO_2_ 101 mmHg	Helmet CPAP group (*n* = 55)	Face mask CPAP group (*n* = 57)	Helmet CPAP 29% Face mask CPAP 59% [95%CI]	In-hospital mortality: Helmet CPAP = 18% [95% CI] Face mask CPAP = 25% [95% CI]	Helmet CPAP compared to Facemask CPAP reduces the endotracheal intubation rate among COVID-19 patients	The use of Helmet CPAP compared to Facemask CPAP reduces the in-hospital mortality rate among COVID-19 patients

*FiO*_*2*_ fraction of inspired oxygen, *PaO*_*2*_ partial pressure of arterial oxygen, *SpO*_*2*_ peripheral capillary oxygen saturation, *HFNO* high-flow nasal oxygen, *NIV* non-invasive ventilation, *CPAP* continuous positive end-expiratory pressure, *AHRF* acute hypoxemic respiratory failure, *ARDS* acute respiratory distress syndrome, *ETI* endotracheal intubation, *BMI* Body Mass Index, *ICU* intensive care unit

### Helmet vs. standard oxygen

In a small trial, helmet CPAP reduced intubation rate (15% vs. 63%) and mortality (5% vs. 40%, 20% when rescue NIV was used in the low-flow oxygen group) in patients with community-acquired pneumonia, compared to conventional oxygen therapy [86].

In a recent meta-analysis by Ferreyro et al. hypothesized the superiority of helmet support over standard oxygen therapy in AHRF: helmet support showed the most significant improvements in mortality (RR 0.40 [0.24–0.63], absolute risk difference − 0.19 [− 0.37 to − 0.09], low certainty of evidence) and intubation rate (RR 0.26 [0.14–0.46], absolute risk difference − 0.32 [− 0.60 to − 0.16], low certainty of evidence) [2]. This meta-analysis included four randomized trials directly comparing helmet CPAP to low-flow oxygen. In addition, facemask NIV showed a lower risk of mortality (RR 0.83 [0–68–0.99], absolute risk difference − 0.06 [− 0.15 to − 0.01, moderate certainty of evidence]) and intubation rates (RR 0.76 [0.62–0.90], absolute risk difference − 0.12 [− 0.25 to − 0.05], moderate certainty) compared to low flow oxygen. These findings are based on an analysis of 13 randomized trials comparing facemask NIV vs. standard oxygen therapy. Interestingly, the beneficial effect of facemask NIV on mortality as compared to standard oxygen was no longer significant when considering patients with more severe disease (PaO_2_/FiO_2_ratio < 200 mmHg), whereas it remained significant for helmet NIV across all degrees of hypoxemia.

### Helmet vs. high flow nasal oxygen

In recent years, there has been significant interest in high flow nasal oxygen as an alternative method to noninvasively manage AHRF. High-flow nasal oxygen provides small, variable amounts of PEEP (2–5 cmH_2_O), anatomical dead space clearance, and an inspiratory flow capable of matching the peak inspiratory flow of a hypoxemic patient, an important advantage over conventional low-flow oxygen therapy devices [87–89]. As a result, HFNC reduces inspiratory effort and improves oxygenation when compared to low-flow oxygen therapy, and its use has become very common in several clinical settings [90–92].

A seminal randomized trial reported that patients with moderate-to-severe AHRF had both lower intubation and mortality rates if treated with HFNC, compared to those treated with NIV delivered through face-mask [78].

In a physiologic, helmet NIV was shown to improve oxygenation and lower inspiratory effort, compared to high-flow nasal oxygen. The most beneficial effects by helmet NIV was observed among most severely hypoxemic patients and those exhibiting intense inspiratory effort (> 10 cmH_2_O) with high-flow nasal oxygen [62].

In the meta-analysis by Ferreyro et al. [2], helmet NIV was associated with decreased mortality (RR 0.46 [0.26–0.80]; absolute risk difference − 0.15 [− 0.34 to − 0.05]; low certainty) and risk of intubation (RR 0.35 [0.18–0.66]; absolute risk difference − 0.20 [− 0.43 to − 0.08]; low certainty) when compared to high-flow oxygen, although no randomized trials directly comparing these two interfaces were included in the metanalysis.

Recently, a multicenter, randomized trial compared early continuous treatment with helmet NIV followed by high-flow nasal oxygen vs. high-flow nasal oxygen on days free of respiratory support in patients with COVID-19 and moderate to severe hypoxemic respiratory failure [17]. This first head-to-head comparison between these two promising techniques demonstrated no difference in respiratory support free days at 28 days. However, helmet NIV was associated with a reduction in the rate of endotracheal intubation in comparison with high-flow nasal oxygen (30% vs. 51%), with an absolute risk reduction of 21% (95% CI 3–38%) and an unadjusted odds ratio of 0.41 (95% CI 0.18–0.89; *P* = 0.03), with no significant effect on mortality. Treatment with helmet NIV was associated with an increased number of days free of invasive ventilation at 28 days from randomization. Patients in the helmet NIV group experienced less dyspnea, improved gas exchange values, with increased discomfort as compared with high-flow nasal oxygen. The most significant clinical benefit of helmet NIV over high-flow nasal oxygen was observed in patients exhibiting hypocapnia before treatment start, which may identify the sub-population with the most dysregulated inspiratory effort [93].

### Helmet vs. facemask NIV

In a matched-control pilot trial in early 2000s, helmet was as effective as the conventional facemask NIV in improving oxygenation and avoiding intubation with better patient comfort and fewer complications (skin necrosis, gastric distension and eye irritation are unusual with helmet interface) [64].

More recently, a retrospective observational study to assess the differences between patients who succeeded or failed noninvasive respiratory support showed that the use of helmet CPAP was an independent predictor of noninvasive respiratory support success and lower intubation rate when compared with facemask NIV [94].

The most rigorous head-to-head comparison of helmet and facemask NIV comes from a randomized trial by Patel and colleagues [18]: patients with ARDS undergoing facemask NIV for at least 8 h were randomly assigned to continue with the facemask or to switch to helmet interface, to assess if helmet NIV could reduce intubation rate and improve outcome. The trial was interrupted after the first interim analysis for efficacy, as helmet use was associated with a significant reduction in the intubation rate (18% with helmet vs. 61% with facemask). Furthermore, helmet NIV was associated with increased ventilator-free days, shorter ICU length of stay and lower hospital and 90-day mortality. In addition, the 1-year follow-up study showed that patients in the helmet group were more likely to be functionally independent, showing a lower incidence of ICU-acquired weakness [3].

A recent non-randomized study confirmed the possible superiority of helmet over facemasks for delivering CPAP in the specific population of COVID-19 patients: use of helmet allowed prolonged treatments with higher PEEP, and was associated with lower rate of intubation and improved survival [95].

Three meta-analyses including studies comparing helmet with facemask NIV in patients with acute respiratory failure confirmed a possible clinical benefit by helmet support [2, 16, 96].

In the network meta-analysis by Ferreyro and colleagues [2], helmet NIV was associated with significantly reduced mortality (RR 0.48 [0.29–0.76]; absolute risk difference − 0.13 [− 0.27 to − 0.05]; low certainty) and risk of endotracheal intubation (RR 0.35 [0.19–0.61]; absolute risk difference − 0.20 [− 0.40 to − 0.09]; low certainty) when compared to facemask NIV.

### Immunocompromised patients

Theoretically, avoidance of intubation is particularly important in immunocompromised patients, for whom respiratory complications are a predominant cause of morbidity and mortality. Squadrone et al. showed that early helmet CPAP in immunosuppressed patients, when compared to standard oxygen, may prevent evolution to ARDS requiring ventilatory support and ICU admission [97]. These results, however, were not confirmed by two recent larger multicentre studies: in immunocompromised patients with AHRF, facemask NIV did not reduce the rate of intubation nor improved clinical outcome as compared to high-flow or low-flow oxygen [98, 99]. It is possible that the helmet interface might be more effective than facemask NIV in immunocompromised patients as well, as suggested by a case–control study conducted by Rocco and colleagues, that compared helmet and facemask NIV in immunocompromised AHRF [100]. However, current evidence does not support a different strategy among immunocompromised patients, since underlying reasons and purposes are similar.

## Conclusions

Noninvasive respiratory support is playing an increasingly important role in the management of patients with severe AHRF. Helmet support may enhance tolerability with greater physiological effectiveness than other noninvasive oxygenation strategies in patients with moderate-to-severe hypoxemia. This is attributable to its ability to deliver higher levels of PEEP for prolonged periods of time with good comfort, which may improve outcomes by improving oxygenation, relieving dyspnea and preventing self-inflicted lung injury and diaphragm injury.

Clinically, helmet support appears to be an effective tool to manage AHRF, especially in patients with the most severe oxygenation impairment. In these patients, helmet NIV could even reduce need for endotracheal intubation, but further research is warranted to confirm findings from preliminary randomized studies and to discriminate the effect of helmet CPAP and NIV. Currently, the lack of confidence with this and technique and the absence of conclusive data regarding its efficacy render helmet use limited to specific settings, with expert and trained personnel. As per any other noninvasive oxygenation strategy, careful monitoring of the patient remains paramount to avoid delays in intubation and institution of protective ventilation.

## Data Availability

Not applicable.
